# Micromask Lithography for Cheap and Fast 2D Materials Microstructures Fabrication

**DOI:** 10.3390/mi12080850

**Published:** 2021-07-21

**Authors:** Mikhail V. Pugachev, Aliaksandr I. Duleba, Arslan A. Galiullin, Aleksandr Y. Kuntsevich

**Affiliations:** 1 P.N. Lebedev Physical Institute of the Russian Academy of Science, 119991 Moscow, Russia; Pugachev.MV@phystech.edu (M.V.P.); Dulebo.AI@phystech.edu (A.I.D.); 2 Department of Physics, National Research University The Higher School of Economics, 101000 Moscow, Russia; aagaliullin_1@edu.hse.ru

**Keywords:** lithography, mask lithography, Van der Waals heterostructures, 2D materials, nanoelectronics, 42.82.Cr

## Abstract

The fast and precise fabrication of micro-devices based on single flakes of novel 2D materials and stacked heterostructures is vital for exploration of novel functionalities. In this paper, we demonstrate a fast high-resolution contact mask lithography through a simple upgrade of metallographic optical microscope. Suggested kit for the micromask lithography is compact and easily compatible with a glove box, thus being suitable for a wide range of air-unstable materials. The shadow masks could be either ordered commercially or fabricated in a laboratory using a beam lithography. The processes of the mask alignment and the resist exposure take a few minutes and provide a micrometer resolution. With the total price of the kit components around USD 200, our approach would be convenient for laboratories with the limited access to commercial lithographic systems.

## 1. Introduction

Two-dimensional materials and Van der Waals heterostructures attract a great interest due to novelty of the properties with respect to traditional 3D materials. Almost all phenomena being made atomically flat revolutionize the corresponding scientific area, e.g., optoelectronics [1], magnetism [2] and superconductivity [3]. More importantly, these materials become the elements of the future devices, e.g., chemical sensors [4], radiation detectors [5], LEDs [6], qubits [7], etc.

Practically, the majority of the novel 2D material-based device prototypes are built up from micrometer sized flakes placed on ∼1 × 1 ${\mathrm{cm}}^{2}$ substrates. The flakes are typically obtained by mechanical exfoliation method [8,9,10], that is applicable to most of layered materials and gives the highest quality 2D crystals. The other fabrication methods, e.g., liquid exfoliation [11] or CVD growth [12], may also produce rather small flakes.

Lithography allows us to pattern mesa-structures, contacts, gate electrodes etc. It is an obligatory stage of the device fabrication. There are many types of lithography, e.g., scanning probe lithography [13], nano-imprint [14], interference lithography [15], stencil mask lithography [16] and others. The majority of these methods are too complicated for 2D materials-related problems. Most of the lithographical processes with microflakes are, therefore, performed using either beam (electron or laser) or mask optical lithography.

Beam lithography is rather expensive (the typical costs of the laser writers and the electron beam lithography machines are ∼USD 100,000 and ∼USD 1,000,000, respectively), time-consuming and requires a fabrication of a system of the marks. The contact mask optical lithography is usually performed with a mask aligner. It is a rather fast process. Commercial mask aligners are designed for $2^{\prime \prime }$–$6^{\prime \prime }$ diameter substrates and include high mechanical accuracy positioning system and sophisticated optics for uniform exposure. These elements are necessary for submicron precision over the whole wafer area and determine high cost (∼USD 100,000) and size of the typical mask aligners [17,18]. Thus, a lithography could be a bottleneck in the 2D materials laboratories.

Another feature of 2D materials is the degradation of almost all of them (except for graphene and hBN) on the air [19,20]. To overcome this issue the time of the air exposure is minimized and the encapsulation between hBN layers in the inert atmosphere is used [20,21,22]. Recently several set-ups were built with lithographers placed into the inert atmosphere gloveboxes [23,24]. Such a combination makes lithography even more expensive.

The existing compact low-cost lithography systems lack for either an alignment option [25,26] or a μm resolution [27,28] required for 2D materials.

This research suggests a solution of the abovementioned problems within a cheap, compact and inert atmosphere-integrable optical mask lithography system. The installation is based on an optical microscope, a manual XYZR stage, an UV-diode and exploits ∼1 × 1 ${\mathrm{cm}}^{2}$ size shadow masks. We demonstrate applicability of the system for 2D materials and other microstructures. The suggested approach paves an alternative to high-priced conventional lithography for microstructures and 2D materials laboratories.

## 2. Materials and Methods

### 2.1. Description of the Installation

**Figure 1:**
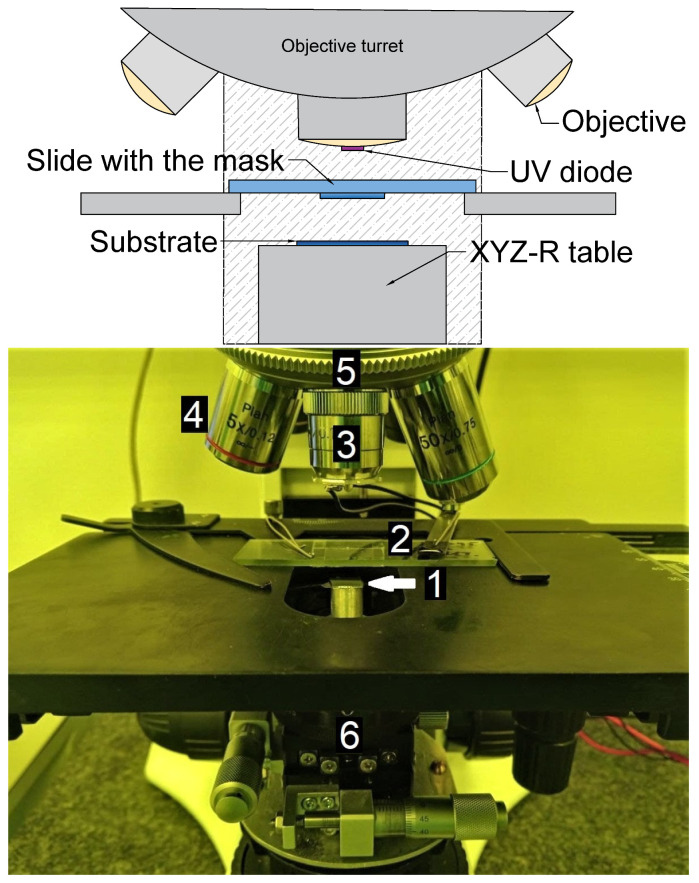
A scheme (**top**) and a photo (**bottom**) of the alignment and exposure microscope kit: 1—the sample fixed to the XYZR stage, 2—mask, 3—UV diode in the lens slot, 4—5× lens, 5—microscope turret, 6—XYZR stage.

Figure 1 shows a scheme and a photograph of the setup. A kit to a metallographic optical microscope (with illumination from the top) includes an XYZR stage, located on the microscope bottom illumination condenser platform, and an UV-diode, located on the objective turret instead of one of the objectives. The substrate is taped to the XYZR stage. The chromium mask is attached to the underside of the microscope slide with the metal-coated side facing the substrate. The microscope slide is fixed in the standard microscope slide position. The mask is positioned with respect to the substrate in the focus of the objective using both the slide movement and the XYZR movement of the substrate.

### 2.2. Mask Fabrication

**Figure 2:**
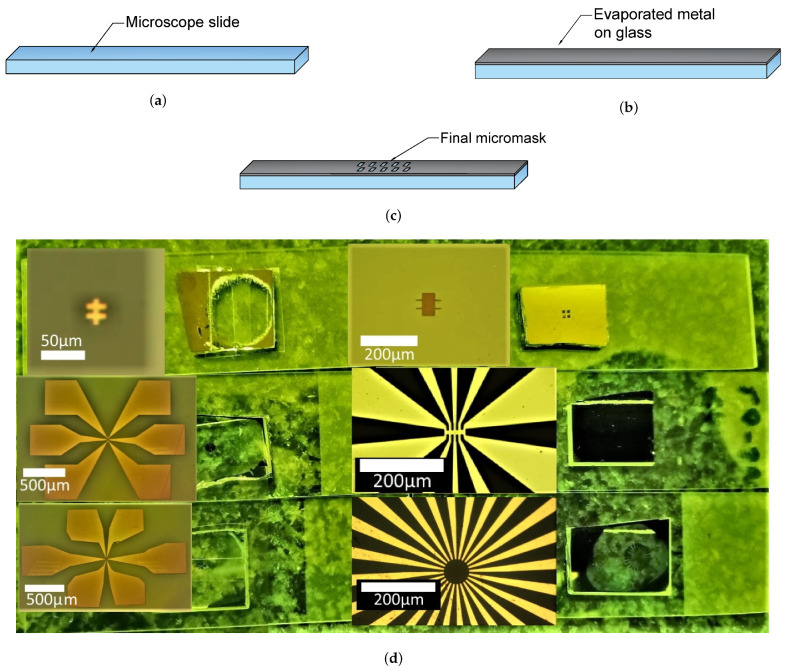
Fabrication of masks based on a microscope glass slide. (**a**) glass; (**b**) evaporated chromium layer on the slide; (**c**) etched mask after optical lithography; (**d**) some of our masks for mesa-structures and contacts.

In this section, we describe how to make a mask in the lab. We use a free version of the KLayout software for drawing the design of the mask in .DXF or .GDS format. Microscope glass slide is used as a carrier (Figure 2a). After cleaning the glass in acetone, isopropanol and deionized water we deposit (resistive heating in $10^{-5}$ Torr vacuum) around 100 nm of chromium (Figure 2b). Then, we spin-coat it with the resist (HS-1512, 1 min, 4 k rpm), bake the resist (50 s, 100 °C). We expose the design of the mask in the resist using the laser-beam lithographer μPG 101 by Heidelberg and develop it (AZ-326 MIF by Microchemicals). Then, we etch the metal (solution Ceric ammonium nitrate: perchloric acid: $H_{2}$O = 10.9%: 4.25%: 84.85% [29]) and remove the rest of the resist with acetone (Figure 2c). A prepared library of the masks (see example in Figure 2d) allows us to make lithography on the samples of various shape and size without making a new unique mask each time. Similar ideas could be found in Ref. [30], where a set of masks with alignment markers of the special shape was suggested.

### 2.3. Lithography Procedure

**Figure 3:**
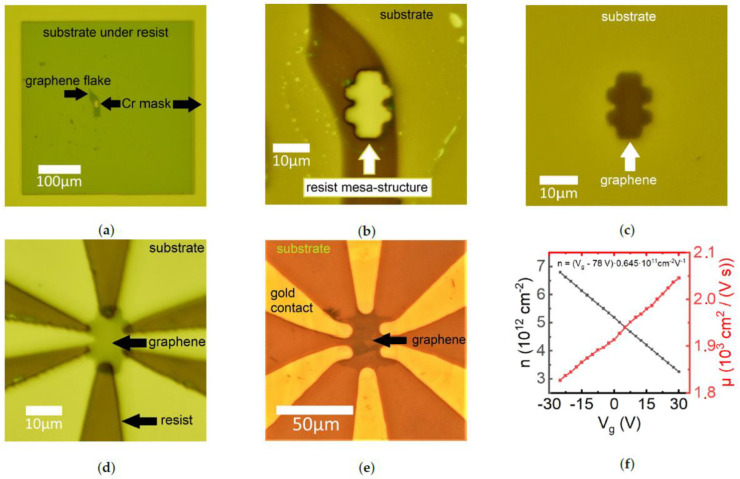
Lithography fabrication stages and examples. Panels (**a**–**d**) show the mesa-fabrication stages on a few-layer graphene flake. The length of the Hall-bar structure is 18 μm. (**a**) Alignment of the mesa mask with 40 μm length graphene flake; (**b**) after the resist developing; (**c**) mesa-structure after the plasma-etching and resist removal; (**d**) micromask-patterned resist for the contacts; (**e**) example of the 50 μm length graphene monolayer Hall-bar mesa-structure with top- evaporated gold contacts, (**f**) Hole density and mobility measured from the Hall effect in the graphene sample at 4 K.

The photos of the stages and the results of the lithography on few-layer graphene samples are shown in Figure 3a–e. We used natural graphite and LLE method of mechanical exfoliation [31] to insulate mono- and few layers of graphene on oxidized Si (285 nm thermally grown ${\mathrm{SiO}}_{2}$ $4^{\prime \prime }$ wafer from Graphene Supermarket [32]). The Si wafer was heavily doped with boron to provide back-gating at the low temperatures.

Using the long working distance 5–20× magnification plan objective, we focus at the level of the chromium layer. We move the substrate in the XY-plane by microcrews and rotate it for alignment with the mask. Due to sufficient depth of focus both the mask and the substrate could be observed simultaneously without a mechanical contact. Then, we bring them into contact (Figure 3a) using Z-stage. The alignment process is shown in the Supplementary Video S1. After the contact, we switch the turret from the objective to the UV diode and turn the diode on, illuminating the photoresist for 5–10 s (resist HS-1512, 405 nm diode 3 W). After the exposure, the resist is developed with a standard developer (AZ-326 MIF by Microchemicals) (Figure 3b). Then, the subsequent procedures can be performed, e.g., metal evaporation or mesa-etching, see Figure 3c–e.

### 2.4. Sample Characterization Methods

The morphology of the studied flakes was determined using the tapping mode of the NT-MDT Solver P47 AFM. Transport measurements were performed in Cryogenics mini-CFMS setup using standard 4-terminal technique at the 4 K temperature and magnetic fields ±1 T. We used SR830 Lock-ins to set the transport current (10 nA, 13 Hz) and measure two components of the resistance. Gate voltage was swept using Yokogawa GS200 precision voltage source. Resistance $R_{xx}$ and Hall resistance $R_{xy}$ values were obtained from the symmetrization and the antisymmetrization of the measurements at 1 T and −1 T, respectively, in order to compensate for the inevitable small contact misalignments and sample non-uniformity. Carrier density *n* was found as $n=B/(eR_{xy})$, where *e* is the elementary charge and B=1 T is the magnetic field. Carrier mobility μ was found as $\mu =R_{xy}l/\left(R_{xx}w\right)$, where *l* and *w* are the distance between the potential probes and the width of the mesa, respectively.

## 3. Results

We discuss below the obtained graphene mesa-structurized samples and the achieved parameters of the set-up.

Figure 3c,d show a plasma-etched graphene mesa-structure and micromask-patterned resist for the lithography of the contacts, respectively. Figure 3e,f show the examples of the structures with gold contacts obtained by different techniques. In Figure 3e, the whole substrate with graphene monolayer flake was first covered with gold, then the contacts were micromask-patterned, then the mesa was defined using the micromask lithography and the plasma-etching.

To demonstrate that the graphene field effect structure works properly, we show in Figure 3f density and mobility of the carriers (holes) as functions of gate voltage at 4 K. The coefficient between the gate voltage and the carrier density agrees well with the used ${\mathrm{SiO}}_{2}$ thickness. The low value of the mobility ∼2000 ${\mathrm{cm}}^{2}$/(Vs) is due to the fabrication process, and in particular, the monolayer contact to metal etchants. Charge neutrality point position at gate voltage $V_{g}=78$ V is also indicative for the high degree of disorder.

**Figure 4:**
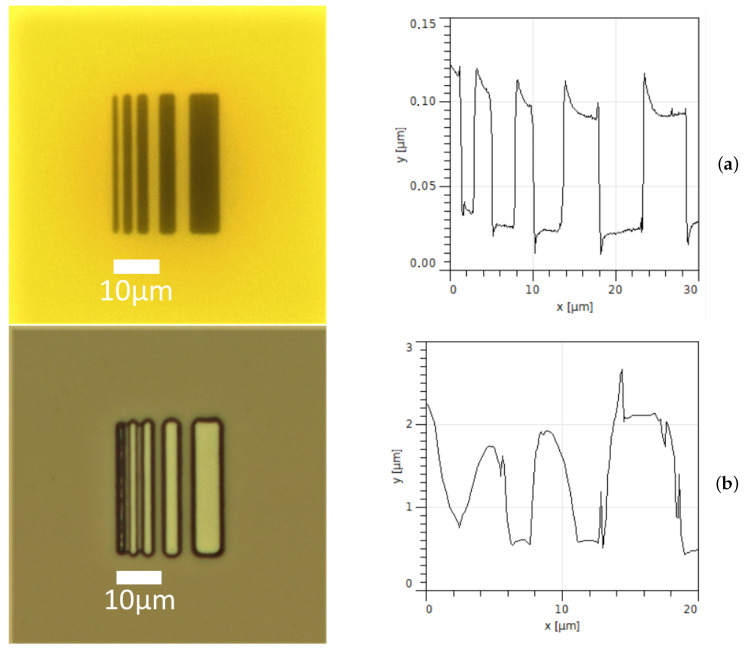
Photo and AFM scan of (**a**) the mask and (**b**) the patterned resist. The narrowest line width and distance between the lines in the mask equal to 1.5 μm.

In order to demonstrate a micrometer resolution, we fabricated a mask with slits down to 1 μm thickness and performed a lithography using this mask, see Figure 4. The resolution was confirmed by AFM scans. Our setup also demonstrates a micrometer accuracy of alignment of the mask and the substrate, see Figure 3b,d–f and Supplementary Materials Video S1.

An important advantage of the micromask lithography with respect to the beam lithography is low time consumption. The alignment of the mask and the substrate requires a few minutes. The exposure of the sample with a UV diode takes 5–10 s, depending on the resist used. The entire lithography process including deposition, baking and developing of the photoresist takes about 10 min.

**Figure 5:**
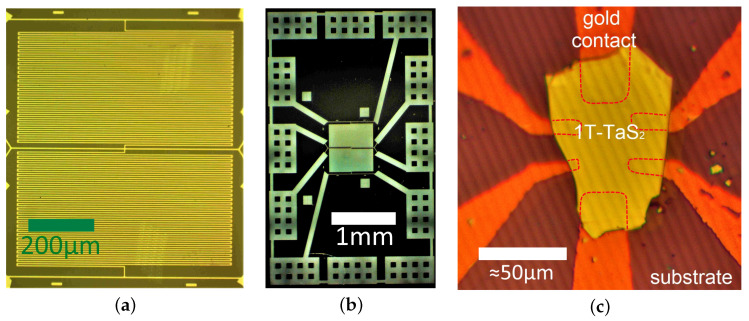
Implementations of the micromask lithography. (**a**) A meander-shaped Al structure on a silicon substrate made by the micromask lithography and the lift-off process; the width of the depicted structure is 850 μm, the thickness of the neighbouring lines and the distance between them are 4 μm. (**b**) Dark-field image of the shadow mask used to produce the meander and contact pads for it; the width of the structure is ≈4 mm. (**c**) A microflake of the electrically switchable material 1T-${\mathrm{TaS}}_{2}$ placed on the micromask-fabricated contact electrodes using the transfer machine.

The setup does not provide a precise alignment at the periphery of the substrate outside the microscope field of view. Nevertheless, the uniformity of the illumination is ensured over the much larger area of ∼1 ${\mathrm{cm}}^{2}$, owing to ∼5 cm LED-to-substrate distance. For example, Figure 5a,b show an Al meander and a macroscopic mask for it. Such a superconducting meander with high kinetic inductance [33,34] can be used, e.g., as a current biasing element with high impedance [35].

The cost of the kit to the microscope consists of the cost of the XYZR stage (in our case ∼USD 100,000), the diode (∼USD 3) and the power supply (∼USD 50), as summarized in Table 1. We also used some screws and standard mechanical parts (not indicated in the table). To reference the prices of some components, that we used for lithography and mask fabrication are also given in Table 1. A clean room with a chemical hood, stable temperature and humidity is highly desirable for the reproducible resist properties. If the ultimate resolution is not the goal, the cost of the process can be dramatically reduced by using a not-so dustless atmosphere and a not-so stable climate-control. In Table 1, we show the typical costs of the ubiquitous resist and developer sufficient for 2–3 micrometer resolution.

The equipment for the resist deposition and baking (spin-coater and heating stage, respectively) could also be purchased within a tiny budget. An expensive professional spin-coater with vacuum sucking of the substrate and programmable control (∼USD 10,000–20,000) can be replaced by a USD 200 PCR centrifuge with the substrate sticking by the double-side scotch-tape. A hot plate with temperature control is also very accessible, as shown in Table 1. All these solutions have been tested in our laboratory. Small items listed below are also quite cheap: acetone, isopropanol, tweezers, nitrile gloves, pipettes, scriber to cut the wafers and glass, and deionized water. Thus, on the basis of our results, we believe that at least 2–3 micrometer-resolution lithography should not be a limiting factor for any 2D materials laboratory.

It is instructive to compare our setup with the other home-made table-top mask lithography machines. In Ref. [36], a mask aligner is built from the sketch. It is much cheaper than the commercial ones (USD 7,500 versus ∼USD 100,000), can work with up to $4^{\prime \prime }$ diameter photomasks and wafers and has slightly lower resolution than the commercial models.

A projection lithographer modification of the optical microscope is reported in Ref. [37]. A photomask is located at the slot of the diaphragm and its image is exposed to the objective focal plane. The system has a resolution of 0.6 μm. Its main disadvantage is a small exposure area of ∼100 μm in diameter that is inconvenient for most of the 2D material-related microstructures.

These works could be compared with the fabrication of contacts and mesa-structures to 2D materials using standard mask aligner with a μm resolution [30]. The comparison is summarized in Table 2.

The prices of our setup and the setup from Ref. [37] in Table 2 we estimated from the cost of the entry level metallographic microscope by AmScope [38].

## 4. Discussion

We discuss below the limitations and the ways to further improve the micromask lithography.

A crucial element that determines the resolution is a photomask. The mask could be either fabricated (as explained in Methods Section) or ordered from numerous suppliers with on-demand design [39,40,41]. Mask of a standard size (e.g., $3^{\prime \prime }$) is too big for the microscope, and could be cut into separate pieces. The typical cost of the mask fabrication is rather affordable, depending on the resolution and can vary between USD 100 and USD 1000. The mask could be produced much more cheaply [42,43] if a μm resolution is not crucial.

The resolution of the photomask can be greatly improved by electronic lithography, and by subsequent using of the mask in combination with ∼260 nm wavelength exposure. Such a wavelength, however, requires a quartz glass slide and also allows us to use UV-sensitive PMMA-based electron resists. Potentially, a nanometer resolution could be achieved via various sophisticated methods of mask production [44,45] essentially exploiting the near-field exposure.

Further development. To assemble Van der Waals heterostructures out of layered crystals, so-called transfer machines are widely used [46,47,48,49]. These setups include a microscope and an XYZR-platform and lack only for a LED to implement the mask lithography. We believe, therefore, that adding a lithography option to these machines is straightforward. Importantly, such transfer machines are placed into the glove boxes with inert atmosphere by many groups routinely [20,21,22], and a lithographer could placed similarly.

Figure 5c demonstrates a combination of micromask lithography drawn contact electrodes and a microflake of 1T-${\mathrm{TaS}}_{2}$ transfered atop using the home-made transfer machine [49]. 1T-${\mathrm{TaS}}_{2}$ is a material with charge-density wave and Mott insulator states including the metastable ones [50]. Electric and optical switching between these states make ${\mathrm{TaS}}_{2}$ microflakes and thin films prospective for memristive applications [51,52].

The XYZR stage can be made motorized and automated, similarly to transfer machines [22], that is very useful for the glovebox operation. Indeed, the resin gloves are not convenient for manual working with the microscope and the XYZR stage.

The alignment marks are not necessary for the objects in the field of view as seen, e.g., from Figure 3. This is an essential advantage, because (i) the time of air exposure is less and (ii) a number of technological steps diminishes. Nevertheless, the described photomask technique is extremely useful for the production of the system of alignment marks on different substrates. Such marks are needed for the beam lithography alignment and also for the location of the flakes in the spatially resolved measurements, see, e.g., photoluminescence [53].

## 5. Conclusions

We demonstrated a cheap, fast and high-resolution contact mask lithography modification of a metallographic microscope that allows us to pattern micro-flakes and other objects placed on ∼1 × 1 ${\mathrm{cm}}^{2}$ substrates. The kit consists of a manipulator, LED, placed in one of the objective turret slots and LED power source. This setup makes a fabrication with a micrometer resolution fast and available to any laboratory. We demonstrate the fabrication of the masks with a micrometer resolution using a beam lithographer. The suggested approach allows us to place microfabrication into the inert atmosphere, which is crucial for the further development of the 2D materials field.

## Figures and Tables

**Table 1:** Components of the micromask-lithography kit.

Component	Price, USD	Website (Accessed on 20 July 2021)
3 W 405 nm LED (10 pcs)	3	Aliexpress
Power supply Gophert NPS1601	47	Aliexpress
XYZR platform	111	Aliexpress
Chromium-coated tungsten rods	17	Aliexpress
Laboratory glass slides, 150 pcs	6	Aliexpress
1000–7000 rpm centrifuge	180	Aliexpress
300 W LCD repair hot plate	40	Aliexpress
Photoresist FP-09-M, 0.5 l	70	frast.ru
Developer UPF-1B, 5 l	20	frast.ru

**Table 2:** Comparison of table-top and home-made mask lithographers.

Parameter	This Work	Projection Lithographer [37]	Mask Aligner [36]	Mask Aligner [30]
Exposure area	1 × 1 cm	0.1 × 0.1 mm	10 × 10 cm	10 × 10 cm
Resolution	∼1.5 μm	0.6 μm	3 μm	∼1 μm
Cost of the machine	USD 200+ microscope USD 1500	UV lamp USD 500+ microscope USD 1500	USD 7500	∼USD 100,000

## Supplementary Materials

The following are available online at https://www.mdpi.com/article/10.3390/mi12080850/s1, Video S1: The alignment process.

## Abbreviations

The following abbreviations are used in this manuscript:

PMMA	poly (methyl methacrylate)
UV	ultraviolet
XYZR stage	a platform, that is controllably positioned along 3 axes and rotated
Z stage	vertical component of XYZR stage
XY stage	horizontal plane of XYZR stage
2D	two dimensional
LED	light-emitting diode
CVD	chemical vapor deposition
LCD	liquid crystal display
PCR	polymerase chain reaction
AFM	atomic-force microscope
LLE	Layer-engineered large-area exfoliation
CFMS	Cryogen Free Measurement System
